# Major concerns and issues in burn survivors in Australia

**DOI:** 10.4103/2321-3868.130192

**Published:** 2014-04-06

**Authors:** Deborah J Dowda, Frank Li

**Affiliations:** 1 Burns Unit, Concord Repatriation General Hospital, Sydney, Australia; 2 Physiotherapy Department, Concord Hospital, Concord, Building 30, Sydney, New South Wales 2139 Australia

**Keywords:** Burns, domains, burn specific health scale, return to work

## Abstract

Burn injury significantly impacts the victim’s long-term quality of life, both physically and psychosocially. This prospective, observational study aimed to assess the physical and psychological health status in adult burn survivors in Sydney Australia using the Burns Specific Health Scale-Brief Version (BSHS-B) questionnaire, together with analysis of the baseline demographic data collected from medical records. A total of 24 adult acute burn victims admitted consecutively to the Burns Unit at Concord Repatriation General Hospital, Sydney, Australia between March 2007 and February 2009 fulfilled the inclusion criteria and participated in the study. The BSHS-B questionnaire (which includes nine domains or subscales) was administered to all 24 participants in person at time of discharge and by mail 6, 12, and 24 months post discharge. By 12 months, 11 participants dropped out and the final analysis was performed on the remaining 13 participants. The analyzed results showed that: 1) Perceived return to work was the only variable that continued to change with time at 12 months after discharge (*P* < 0.01); 2) At 12 months; return to work was significantly correlated with simple functional ability (*P* < 0.05), heat sensitivity (*P* < 0.01), and treatment regimes (*P* < 0.05), but no longer with affect and body image as demonstrated at 6 months. In summary, our findings have shown that the perception of returning to work changes significantly with time post discharge and this perception is affected by certain subscales of the BSHS-B. Given that return to work is one of the most important outcome concerns and issues of recovery for adult burn injury victims and families, it is essential that therapists be aware of the factors influencing return to work and address these factors through a comprehensive rehabilitation program.

## Introduction

A burn injury is an unforeseen event that impacts the person in a variety of ways.[1–3] Physically, burn survivors often suffer from chronic pain and sensory problems, pruritus, and loss of strength.[4–6] Psychosocially, a certain proportion may develop depression and posttraumatic stress disorder after hospital discharge.[7,8] Economically, absence from work after burn injury imposes a considerable economic burden to individuals employed at the time of the burn injury.[4,9,10] Therefore, to these individuals, early return to work is the most demanding goal of treatment and rehabilitation.[11] To achieve this goal, the factors that influence return to work after a burn injury need to be well-understood.

**Table:** 

Access this article online	
**Quick Response Code**:	**Website**: www.burnstrauma.com
	**DOI**: 10.4103/2321-3868.130192

As recently and comprehensively reviewed by Mason and colleagues,[12] 26 peer-reviewed studies published in English since 1970 have attempted to investigate predictors and barriers of returning to work in previously employed burn victims. These studies have identified burn location, burn size, treatment variable, age, pain, psychosocial factors, job factors, and barriers as the major influencing factors of return to work in adult (means age of 33.63 years) individuals surviving a burn injury involving 18.94% of the total body surface area (TBSA) on average. However, none of these reviewed studies is from Sydney, Australia. Given the possible variation in factors that influence return to work after a burn injury in different cultural backgrounds and geographical areas, this prospective observational study was conducted to identify the major predictors of return to work in a sample of previously employed adult acute burn survivors in Sydney, Australia through the burns specific health scale-brief version (BSHS-B)-based assessment of the physical and psychosocial health status.

## Materials and methods

### Participants

Participants of this questionnaire survey study were chosen from acute burn patients admitted to the Burns Unit at Concord Repatriation General Hospital, Sydney, Australia between March 2007 and February 2009. The inclusion criteria were:

1. Age ≥ 18 years;
2. Burn injury involving ≥ 10% of the TBSA; and
3. Hospital stay ≥ 7 days. Patients with severe psychiatric diagnosis or dementia, patients unable to use English to adequately make follow-up communications with the investigators, and prisoners were all excluded.

### Questionnaire survey

The BSHS-B questionnaire was used as an instrument to assess the quality of life of burn victims from the physical, mental, and social aspects. The questions covered 9 specific domains, which fell into 2 subcategories: Physical health status (simple function abilities, hand function, work, heat sensitivity, and treatment regimes) and psychological aspects (affect, body image, sexuality, and interpersonal relationships). The BSHS questionnaire was distributed to and collected from all participants in person at hospital discharge and by mail at 3 months, 6 months, 1 year, and 2 years after discharge. The baseline demographic data were retrieved from the medical records of the participants.

### Statistical analysis

The program Statistical Package for Social Sciences (SPSS, Chicago, IL, USA) was used. Data on subscales of BSHS-B were analyzed by paired *t*-test. Correlations between various subscales of BSHS-B as well as between BSHS-B subscales and the perception of return to work were analyzed by Pearson’s correlation test. *P* < 0.05 was considered as the criterion of statistical significance.

## Results

### Demographic and clinical characteristics

A total of 46 subjects met the inclusion criteria and agreed to participate. However, only 24 completed the initial survey at the time of discharge. Their baseline demographic and clinical characteristics with insurance information are presented in Table 1 in a standard format developed by the New South Wales State-wide Burn Injury Service (NSWSBIS) as a Social Work Data Entry Form 2006. The vast majority (96%) of these 24 subjects were male. On average, they were 43 years of age with one-fifth of the TBSA burned. Both face and hand(s) were injured in approximately 60% of the subjects. Fire accounted for more than 50% of the injuries.

**Table 1:** **Baseline demographic and clinical data presented in the New South Wales State-wide Burn Injury Service (NSWSBIS) social work data entry form 2006**

Total patients		24
	Sex	
	Male	23 (96%)
Female		1 (4%)
Age		43 (21–81) years
TBSA		21% (10–68%)
Average hospital stay		28 (7–75) days
Burn involving		
	Face	15 (63%)
	Hand(s)	14 (58%)
Burn mechanisms		
	Flame	13 (54%)
	Scald	6 (25%)
	Chemical	3 (12.5%)
	others	2 (8.5%)
Grafting required		16 (67%)
Insurance-workers compensation		8 (33.3%)
Occupation		
	Technical/trade	7 (29.2%)
	Labor/farmer	6 (25%)
	Pensioner	4 (16.7%)
	Non-specified	7 (29.2%)

### Scores of BSHS-B subscales at different time points

Presented in Table 2 are scores of BSHS-B subscales at discharge and at 6 and 12 months after discharge. The sub-scale score for simple functional ability at 12 months after discharge was significantly higher than that at discharge (*P* < 0.01). The score for the perception in returning to work at both 6 and 12 months after discharge was significantly higher than that at discharge (*P* < 0.01). There were no significant differences in all other subscales between different time points (*P* > 0.05).

**Table 2:** Mean scores of BSHS-B subscales at discharge, 6 months and 12 months after discharge. A single asterisk (*) indicates a significant difference from the score at discharge within the same a domain at α = 0.01 ( ***n*** **= 13)**

Domain	Discharge	6 months	12 months
Affect	20.93±3.13	20.47±4.55	20.55±3.83
Body image	12.07±4.32	12.93±3.93	12.10±3.25
Heat sensitivity	8.67±5.81	9.08±6.17	9.25±6.17
Hand function	15.85±5.67	18.15±2.94	19.36±1.12
Interpersonal relationship	17.71±3.29	17.79±3.38	18.82±1.72
Simple functional ability	9.07±2.60	10.04±3.27	11.83±0.39*
Sexuality	10.46±2.63	11.62±0.77	10.30±1.77
Treatment regimens	12.20±3.84	11.80±4.59	13.08±3.23
Work	6.08±3.35	9.08±5.47*	11.20±4.94*

### Correlation of work with other BSHS-B subscales

As shown in Table 3, at 6 months after discharge, the perception of return to work was highly correlated with affect (*P* < 0.05), body image (*P* < 0.01), heat sensitivity (*P* < 0.01), and treatment regimens (*P* < 0.01). By 12 months after discharge, perceived return to work remained significantly correlated heat sensitivity and treatment regimens (*P* < 0.05), but no longer with affect and body image (*P* > 0.05), while simple functional ability became significantly correlated with work (*P* < 0.05).

**Table 3:** Correlation coefficients between the perceived return to work and other BSHS-B subscales at 6 and 12 months after discharge. A single (*) and double (**) asterisks indicate significance at α = 0.05 and α = 0.01, respectively ( ***n*** **= 13)**

Domain	6 months	12 months
Affect	0.602*	0.133
Body image	0.7502**	0.405
Heat sensitivity	0.9256**	0.758*
Hand function	0.149	0.538
Interpersonal relationship	0.428	0.03
Simple functional ability	0.275	0.621*
Sexuality	0.163	0.116
Treatment regimens	0.889**	0.625*

### Correlation of work with major demographic and clinical variables

As shown in Table 4, the perception of returning to work was correlated to the length of hospital stay, TBSA burned, and workers’compensation (*P* < 0.05) at 6 months and remained correlated with TBSA burned and workers’compensation (*P* < 0.05) and became correlated with facial injury (*P* < 0.05) at 12 months. Age, hand injury, and occupation were not correlated with the perceived return to work at either time points (*P* > 0.05).

**Table 4:** Correlation coefficients between the perceived return to work and the major demographic and clinical variables at 6 and 12 months after discharge. A single (*) asterisk indicates significance at α = 0.05 ( ***n*** **= 13)**

Domain	6 months	12 months
Age	0.436	0.516
Hand injury	0.482	0.478
Facial injury	0.541	0.620*
Length of stay	0.700*	0.472
Occupation	0.100	0.252
% TBSA	0.684*	0.668*
Workers’ compensation	0.800*	0.452*

## Discussion

Global epidemiologic data indicate that fire-related burns are among the leading causes of disability-adjusted life years lost in developing countries.[13,14] In contrast, most of the adult burn cases in high-income developed countries occur as a result of workplace accidents.[15,16] In agreement with the etiologic characteristics of burn injuries in developed countries,[12,17,18] our analysis showed that out of the 24 burn victims assessed, 13 (54.2%) were technical/trade staff and 3 (12.5%) farmers or other physical laborers and 8 (33.3%) were injured at work [Table 1].

A severe burn injury significantly impacts on all aspects of the quality of life. In this study, we evaluated the impact of burn injury on the survivors from nine aspects using the BSHS-B. At 12 months post discharge, heat sensitivity, simple functional ability, and treatment were all correlated with the quality of life in the burn survivors assessed [Table 2]. This finding strongly supports the profound importance of returning to work for adult burn survivors employed at the time of the injury.[19]

TBSA of burns is the major factor determining the time to return to work post-burn injury.[20] Obviously, the larger the area affected, the longer the length of hospital stay and/or post-discharge physical rehabilitation is needed; it is estimated that in burn victims with affected TBSA between 25% and 40%, the amount of time off work is approximately 6–12 months.[12] In line with this estimation, the average TBSA was 21%. Data around return to work, however, was not available from this questionnaire. Among the other factors affecting the time of return to work assessed in our study, age, hand injury and occupation were all not significant, but facial injury became a more significant influencing factor with time [Table 4]. These observations indicate that adult burn survivors are more concerned with physical and functional impairments in early post-burn stage, but become more concerned with their body image damage when they have physically and functionally recovered to a level that allows them to return to work. The concern over perception of their body image at the time of return to work is understandable; if the injured person is going to return to a job involving face-to-face contacts with customers, in business or public services, for example, scarring on the face may not be seen as necessarily ‘good for business’.

Given the notion that ‘knowledge of impairments that influence occupational outcomes must guide clinical treatment and resource allocation, as well as long-term follow-up and impairment guided intervention’,^1211^ burn therapy and rehabilitation strategies should fully reflect post-burn stage-specific concerns and requirements of adults injured at work. More specifically, to prevent death and restore their physical and functional activities are the priority of early-stage burn management; whereas, cosmetic correction of impaired appearance should be paid sufficient attention.

In summary, through a questionnaire survey using the BSHS-B, we demonstrated that return to work was the most consistent and important concern and the total body area burned and facial injury were among the most influential factors of the time to return to work in a cohort of adult burn survivors in Sydney, Australia. These observations suggest that stage-related concerns should be taken into account in the management and rehabilitation of burn injuries in adult survivors.
